# Supplementary material for: Archaeal TFEα/β is a hybrid of TFIIE and the RNA polymerase III subcomplex hRPC62/39
Source: eLife. 2015 Jun 12;4:e08378. doi: 10.7554/eLife.08378 (PMC4495717; doi:10.7554/eLife.08378)
Supplement: Table 1—source data 1. — Genetic experiments showing that the TFEβ encoding gene Saci_1342 is essential DOI: http://dx.doi.org/10.7554/eLife.08378.007 [file elife08378s001.pdf]

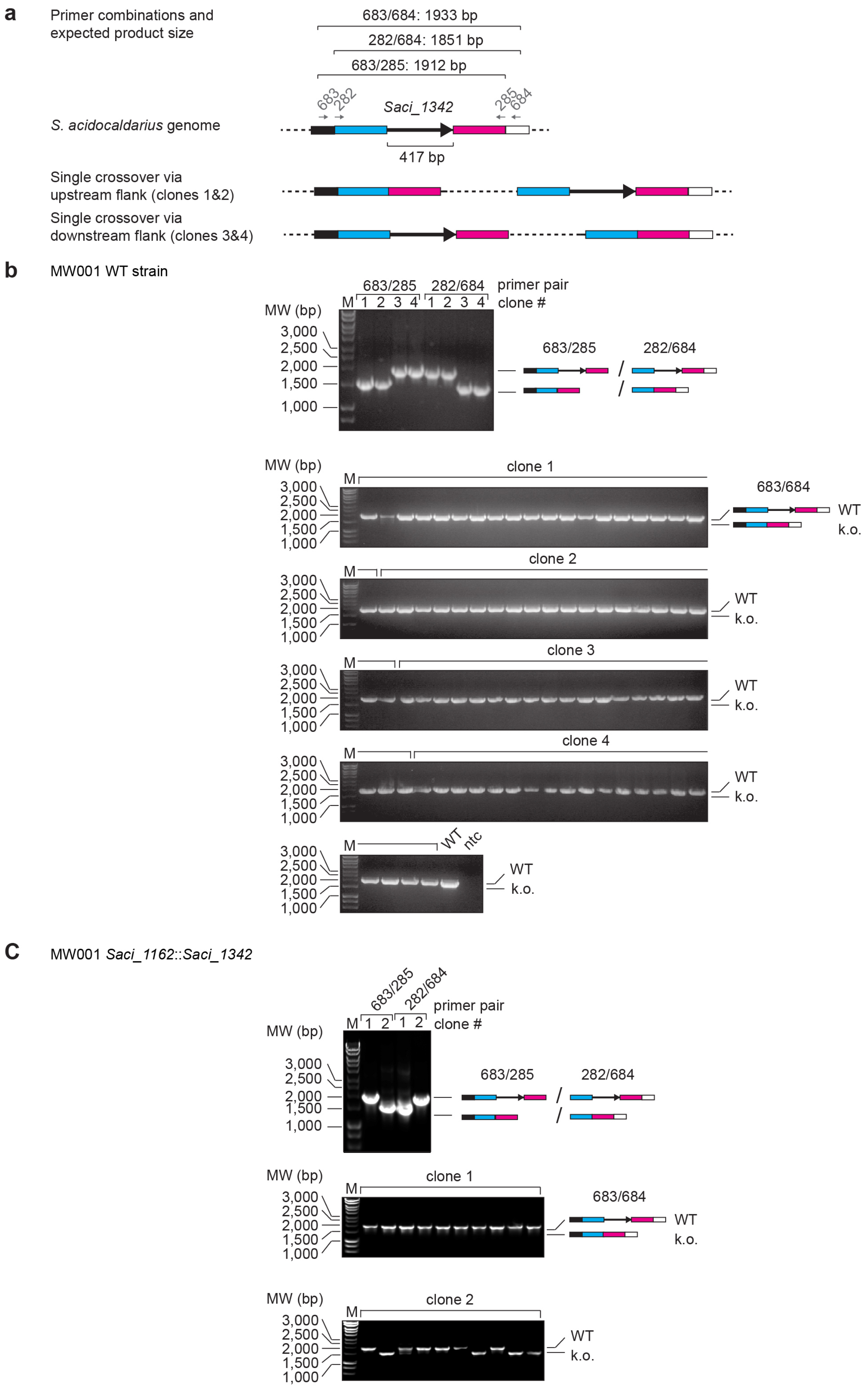

a
Primer combinations and expected product size
683/684: 1933 bp
282/684: 1851 bp
683/285: 1912 bp
683
282
Saci_1342
285
684
S. acidocaldarius genome
417 bp
Single crossover via upstream flank (clones 1&2)
Single crossover via downstream flank (clones 3&4)
b
MW001 WT strain
683/285
282/684
primer pair
clone #
M
1 2 3 4 1 2 3 4
MW (bp)
3,000
2,500
2,000
1,500
1,000
683/285
282/684
clone 1
683/684
WT
k.o.
clone 2
clone 3
clone 4
WT
ntc
C
MW001 Saci_1162::Saci_1342
683/285
282/684
primer pair
clone #
1 2 1 2
clone 1
clone 2

Table 1 – source data 1. (a) Overview of the *Saci_1342* locus in *S. acidocaldarius* MW001. The upstream flank (blue) and the downstream flank (magenta) are indicated as well as the positions targeted by the primers used for PCR analysis of the mutant strains and the size of the respective PCR products (small arrows). The two possible outcomes after integration via single-crossover of circular DNA p1085 bearing a fusion of the upstream and downstream flanks are depicted. **(b)** the upper agarose gel shows the PCR analysis of four colonies after p1085 integration in MW001 WT. The four agarose gels below show the PCR analysis after counterselection with 5-FOA. 80 colonies were tested, 40 of which were derived from p1085 integration via the upstream flanking region (clones 1&2) and 40 via the downstream flanking region (clones 3&4). The expected sizes of PCR products for reverted WT and *Saci_1342* deletion (k.o.) are depicted. All tested clones reverted to WT via reciprocal excision of p1085. A sample with genomic DNA from the genetic background strain MW001 (WT) was included as well as a no-template reaction (ntc) serving as negative control. **(c)** *Saci_1342* deletion in MW001 *Saci_1162*::*Saci_1342*. In this strain the non-essential gene *Saci_1162* was replaced with a second copy of *Saci_1342*. Analysis of the transformants was as described as aboved with 10 clones derived from p1085 integration via the upstream flanking region (clone 2) and 10 via the downstream flanking region (clone1) being analysed. The fact that only upstream flank integration of p1085 allowed for *Saci_1342* deletion may hint at additional restraints.
