# Supplementary material for: Archaeal TFEα/β is a hybrid of TFIIE and the RNA polymerase III subcomplex hRPC62/39
Source: eLife. 2015 Jun 12;4:e08378. doi: 10.7554/eLife.08378 (PMC4495717; doi:10.7554/eLife.08378)
Supplement: Figure 2—source data 1. — DOI: http://dx.doi.org/10.7554/eLife.08378.009 [file elife08378s002.pdf]

Figure 2 – Source data 1 – theoretical and experimentally calculated masses of proteins and protein complexes.

	Protein	Theoretical Mass	Experimental Mass	Mass Error	Mass Difference (Da)	Mass Difference (%)
**Native MS**	TFEα + Zn	21236.5	21236.6	4.4	0.2	0.001
	TFEα + Zn + TFEβ + [4Fe-4S]	36523.5	36530.8	11.9	7.3	0.020
	hRPC62	62489.6	62516.3	11.8	26.7	0.043
	hRPC39	35552.8	35555.7	13.6	2.9	0.008
	hRPC62/39 + [4Fe-4S]	98392.0	98455.5	3.6	63.5	0.064
**Tandem MS**	hRPC39	35552.8	35555.8	0.6	3.0	0.008
	hRPC39 + [4Fe-4S]	35902.4	35905.7	0.2	3.3	0.009
