# Supplementary material for: Archaeal TFEα/β is a hybrid of TFIIE and the RNA polymerase III subcomplex hRPC62/39
Source: eLife. 2015 Jun 12;4:e08378. doi: 10.7554/eLife.08378 (PMC4495717; doi:10.7554/eLife.08378)
Supplement: Supplementary file 1. — List of plasmids generated by restriction enzyme-based cloning. DOI: http://dx.doi.org/10.7554/eLife.08378.022 [file elife08378s003.pdf]

Supplementary file 1 – List of plasmids generated by restriction enzyme-based cloning

Plasmid ID	Backbone	Insert(s)	Restriction sites	Oligonucleotide pairs used for amplification
p988	pET21a+ (Merck Millipore)	*Sso* TFEα	NdeI/EcoRI	FW239/FW240
p1051	pSVA1481 (S.V. Albers, unpublished)	*Sso* TFEβ C-His$_{10}$	BamHI/NcoI	FW280/FW281
p1056	pMJ0503 (Jonuscheit et al., 2003)	*Sso* TFEβ C-His$_{10}$	AvrII/EagI	subcloning from p1051
p1058	pSVA406 (Wagner et al., 2012)	Overlap extension PCR product fusing the upstream and downstream flanks of *Saci_1342*	NcoI/BamHI	FW282/FW283 and FW284/FW285
p1071	pET21a+ (Merck Millipore)	*Sso* TFEα Δ111-178	NdeI/EcoRI	FW239/FW333
p1076	pET21a+ (Merck Millipore)	*Sso* TFEα and *Sso tfeβ* C-His$_6$	NdeI/EcoRI/XhoI	FW239/FW240 and FW242/FW279
p1077	pRSF-1b (Merck Millipore)	*Sso* TFEβ C-His$_6$	NcoI/AvrII	FW280/FW336 (amplified from p1050)
p1087	pET21a+ (Merck Millipore)	*Sso* TFB (Sso0446) C-His$_6$	NdeI/XhoI	FW337/FW338
p1092	pRSF-1b (Merck Millipore)/p1077	*Sso* TFEβ C-His$_6$ Δ74-125	NdeI/XhoI	FW280/FW347
p1094	pRSF-1b (Merck Millipore)/p1077	*Sso* TFEβ C-His$_6$ Δ85-125	NdeI/XhoI	FW280/FW349
p1107	pET21a+ (Merck Millipore)	*Sso* TFEα Δ1-110	NdeI/EcoRI	FW387/FW240
p1108	pET21a+ (Merck Millipore)	*Sso* TFEα Δ148-178	NdeI/EcoRI	FW239/FW388
p1110	pRSF-1b (Merck Millipore)/p1077	*Sso* TFEβ C-His$_6$ Δ1-73	NcoI+ BspHI (compatible ends)/XhoI	FW396/FW279
p1111	pRSF-1b (Merck	*Sso* TFEβ C-His$_6$ Δ1-84	NcoI+ BspHI	FW397/FW279

	Millipore)/p1077		(compatible ends)/XhoI	
p1112	pSVA406 (Wagner et al., 2012)	Overlap extension PCR product fusing the upstream and downstream flanks of *Saci_1162 to the Saci_1342* including 100 nt upstream of its predicted translation start site	PstI/BamHI	FW326/FW327, FW328/FW329 and FW330/FW331
p1121	pET21a+ (Merck Millipore)	*Sso* TBP	NdeI/XhoI	FW449/340
p1159	pET21a+ (Merck Millipore)	N-His$_6$ hRPC62 and hRPC39	NdeI/EcoRI/XhoI	FW569/FW570 and FW566/FW567
p1172	pRSF-1b (Merck Millipore)/p1077	recombinant RNAP clamp fusion of Rpo2 1055-1117, Rpo1’ 4-315, and Rpo1” 340-377 C-His$_6$	NcoI+ BspHI (compatible ends)/XhoI	FW673/FW436/FW437 /FW438/FW439/FW44 0 (Overlap extension PCR)
pSVA143	pMZ1 (Zolghadr et al., 2007)	*Sso* Rpo8 C-His$_{10}$ (Sso0277)	NcoI/BamHI	509/510
pSVA158	pMJ0503 (Jonuscheit et al. , 2003)	*Sso* Rpo8 C-His$_{10}$ (Sso0277)	BlgI/EagI	subcloning from pSVA143

Additional reference:

Zolghadr, B., S. Weber, Z. Szabo, A. J. Driessen and S. V. Albers (2007). "Identification of a system required for the functional surface localization of sugar binding proteins with class III signal peptides in Sulfolobus solfataricus." Mol Microbiol **64**(3): 795-806.
