# Supplementary material for: Archaeal TFEα/β is a hybrid of TFIIE and the RNA polymerase III subcomplex hRPC62/39
Source: eLife. 2015 Jun 12;4:e08378. doi: 10.7554/eLife.08378 (PMC4495717; doi:10.7554/eLife.08378)
Supplement: Supplementary file 2. — List of plasmids generated by PCR-based Site-directed mutagenesis. DOI: http://dx.doi.org/10.7554/eLife.08378.023 [file elife08378s004.pdf]

**Supplementary file 2 – List of plasmids generated by PCR-based Site-directed mutagenesis**

| Plasmid ID | Template | Mutation | Oligonucleotides |
|---|---|---|---|
| p1072 | p988 | *Sso* TFEα K46E | FW322/FW323 |
| p1073 | p988 | *Sso* TFEα R51E/K52E | FW324/FW325 |
| p1074 | p988 | *Sso* TFEα D49T | FW334/FW335 |
| p1090 | p988 | *Sso* TFEα Δ46-52 | FW345/FW346 |
| p1118 | p1077 | *Sso* TFEβ C-$His_6$ C112S | FW443/FW444 |
| p1120 | p1077 | *Sso* TFEβ C-$His_6$ C101S | FW447/FW448 |
| p1173 | p1077 | *Sso* TFEβ C-$His_6$ K64E | FW669/FW670 |
| p1174 | p1077 | *Sso* TFEβ C-$His_6$ K56E/K57E | FW671/FW672 |
| p1175 | p988 | *Sso* TFEα C117S | FW667/FW668 |
| p1181 | p988 | *Sso* TFEα Δ114-147 | FW665/FW666 |
| p1200 | p1077 | *Sso* TFEβ C-$His_6$ C92S | FW733/FW734 |
| p1201 | p1077 | *Sso* TFEβ C-$His_6$ C95S | FW735/FW736 |
