# Supplementary material for: Archaeal TFEα/β is a hybrid of TFIIE and the RNA polymerase III subcomplex hRPC62/39
Source: eLife. 2015 Jun 12;4:e08378. doi: 10.7554/eLife.08378 (PMC4495717; doi:10.7554/eLife.08378)
Supplement: Supplementary file 3. — List of oligonucleotides used in this study. DOI: http://dx.doi.org/10.7554/eLife.08378.024 [file elife08378s005.pdf]

Supplementary file 3 – List of oligonucleotides used in this study*

Primer ID	Name	Sequence
FW239	*Sso* TFEα NdeI fw	GGCATATGGTTAACGCAGAGGATCTGTTTATC
FW240	*Sso* TFEα stp EcoRI rv	GGGAATTCTCAATGATTTTTATTAGCTCCAAG
FW242	*Sso* TFEβ RBS EcoRI fw	GGGAATTCAG**GAGG**TAATTAAATATGGAATCAGATTCAACAAGTAATTATGAAGC
FW279	*Sso* TFEβ XhoI rv	GGCTCGAGAGATAAAGCTTTTTCTATGATCCAGC
FW280	*Sso* TFEβ NcoI fw	CCCCCATGGAATCAGATTCAACAAGTAATT
FW281	*Sso* TFEβ BamHI rv	GGCGGATCCAGATAAAGCTTTTTCTATGATCCAGC
FW282	*Saci_1342* upstream flank NcoI fw	CCATGGAAGAACAGGTTCTCCTCTAACAGC
FW283	*Saci_1342* upstream flank rv	CACTAGTTAAGATATTTTCCCCATTTATTTACTTTATTTCC
FW284	*Saci_1342* downstream flank fw	AAATAAATGGGGAAAATATCTTAACTAGTGTTTCTGCTGGATAAC
FW285	*Saci_1342* downstream flank BamHI rv	GGATCCACGTTGCTATAACTGCTACAGATCTT
FW322	*Sso* TFEα K46E fw	CAGTTGAATATAGAAGTTAATGATGTAAGGAAG
FW323	*Sso* TFEα K46E rv	CTTACATCATTAACTTCTATATTCAACTGATTCGC
FW324	*Sso* TFEα R51E/K52E fw	GTTAATGATGTAGAGGAGAAATTAAATTTACTAGAGGAG
FW325	*Sso* TFEα R51E/K52E rv	GTAAATTTAATTTCTCCTCTACATCATTAACTTTTATATTCAAC
FW326	*Saci_1162* upstream flank PstI fw	GGCCGGCTGCAGATATTATGACATTTTGTTTCAGGAGC
FW327	*Saci_1162* upstream flank rv	ATGCTCAAACTATAGGACTACTTTCATAAATAAAAATAGATATTCAA
FW328	*Saci_1342* promoter fw	GAAAGTAGTCCTATAGTTTGAGCATAACAGAAGTAACAATAAG
FW329	*Saci_1342* rv	TAAATTATGCTATCCAGCAGAAACACTAGTTAAGATAT
FW330	*Saci_1162* downstream flank fw	TCTGCTGGATAGCATAATTTATAACTAAAATATAAGGTAAAAAA
FW331	*Saci_1162* downstream flank BamHI rv	GCGCGCGGATCCGATGTGGATGACAAAGAGCACT
FW333	*Sso* TFEα Δ111-178 stp EcoRI rv	GCGGGAATTCTCATTTCTCATATTCCAATCTTGTC
FW334	*Sso* TFEα D49T fw	GAATATAAAAGTTAATACGGTAAGGAAGAAATTAAATTTACT
FW335	*Sso* TFEα D49T rv	TAATTTCTTCCTTACCGTATTAACTTTTATATTCAACTGAT
FW336	pET21a+ AvrII rv	GGCGCCTAGGCAGCTTCCTTTCGGGCTT
FW337	*Sso* TFB NdeI fw	GGCGCATATGTTGTATTTGTCTGAAGAAAATAAATCC

FW338	*Sso* TFB XhoI rv	GCGGCTCGAGTTGAGTAGGTATTGATATTTTTAGCTCTTG
FW340	*Sso* TBP stp XhoI rv	GCGGCTCGAGTTAGAGCTCTAACTCTTCCTCTTCCTC
FW345	*Sso* TFEα Δ46-52 fw	GGCGGTGGCGGTAAATTAAATTTACTAGAGGAGCAAGGT
FW346	*Sso* TFEα Δ46-52 rv	TATATTCAACTGATTCGCTATTTCTTC
FW347	*Sso* TFEβ Δ74-125 XhoI rv	GCGGCTCGAGATCAACAGCAAATAGTTTTACTATATTTTT
FW349	*Sso* TFEβ Δ85-125 XhoI rv	GCGGCTCGAGAAGATTTATATAAATGTTTATATTATAATTATTATCAACA
FW387	*Sso* TFEα Δ1-110 NdeI fw	GGCGCATATGAATAATACGTTCTTTATATGCCCTCAA
FW388	*Sso* TFEα Δ148-178 stp EcoRI rv	GCGGGAATTCTCAATCATAATAGGTTAGTTGCGATCCA
FW391	*Sso* TFEβ Δ115-125XhoIrv	GCGGCTCGAGTTTACTGCAAGTTTGTGGACTTACA
FW396	*Sso* TFEβ Δ1-73 BspHI fw	GGCGTCATGAATAATTATAATATAAACATTTATATAAATCTTAATGGTT
FW397	*Sso* TFEβ Δ1-84 BspHI fw	GGCGTCATGAATGGTTTAGATGAAATTCCTTGC
FW436	*Sso* Rpo2 1055-1117 rv	TCCTTTTATATTCTTACCGCCATCCTCCAAAACTAACCTAGGTGAG
FW437	*Sso* Rpo1’ 4-315 fw	TTAGTTTTGGAGGATGGCGGTAAGAATATAAAAGGAATAAAGTTTGGAAT
FW438	*Sso* Rpo1’ 4-315 rv	ACCCGCGCCGCTGCCCGCGCCACCAGATAAATTACCTCTAAATCTACCTTC
FW439	*Sso* Rpo1” 340-377 fw	GGCGCGGGCAGCGGCGCGGGTAGCGGTAGTGTATTAG CAAGAGCT
FW440	*Sso* Rpo1” 340-377 XhoI rv	GCCGCTCGAGATGACCAATTATAATGTTTTCTACTACACCT
FW443	*Sso* TFEβ C112S fw	GTAAGTCCACAAACTTCCAGTAAATTATCCGGCTGGAT
FW444	*Sso* TFEβ C112S rv	GCCGGATAATTTACTGGAAGTTTGTGGACTTACATGTAT
FW447	*Sso* TFEβ C101S fw	GTAAAAGCTTAACCAAATCTGGAAATGGAATACATGTAAG
FW448	*Sso* TFEβ C101S rv	GTATTCCATTTCCAGATTTGGTTAAGCTTTTACAACTT
FW449	*Sso* TBP *NdeI* fw	GGCGCATATGTCCAATTCAGCGGTATCGTAT
FW566	hC39 RBS EcoRI fw	GGCGAATTCAG**GAGG**TAATTAAATATGGCAGAAGTGAAAGTGAAAGT
FW567	hC39 stp XhoI rv	GCGCTCGAGTTAAAATTCCAGCCATTCGG
FW569	hC62 N-His NdeI fw	GCGCATATGGCACACCATCACCAC
FW570	hC62 stp EcoRI rev	GGCGAATTCTTATTGGCGTTTCATCGTACA
FW642	Promoter SSV1 T6 C-less fw	GATTGATAGAGTAAAGTTTAAATACTTATATAGATAGAGTATAGATAGAGGGTATG GAAGGAGAATATAATTGAG
FW643	C-less cassette rv	TCATTCACTCTCATCCCCTCTT
FW665	*Sso* TFEα Δ114-147 fw	GGCGGTGGCGGTACGGATAAAATTAAGTCCTTCTT
FW666	*Sso* TFEα Δ114-147 rv	CGTATTATTTTTCTCATATTCCAA
FW667	*Sso* TFEα C117S fw	AATACGTTCTTTATAAGCCCTCAAGATAATAGTAGA
FW668	*Sso* TFEα C117S rv	ATCTTGAGGGCTTATAAAGAACGTATTATTTTTCTCA
FW669	*Sso* TFEβ K64E fw	GTTTTACTATATTTTCCCCATTTTCTTTCACTGC
FW670	*Sso* TFEβ K64E rv	GAAAGAAAATGGGGAAAATATAGTAAAACTATTTGCTGT

FW671	*Sso* TFEβ K56E/K57E fw	GAAGAAACTAATTATTGAAGAAGCAGTGAAAGAAAATGGG
FW672	*Sso* TFEβ K56E/K57E rv	CTTTCACTGCTTCTTCAATAATTAGTTTCTTCTCGACT
FW673	*Sso* Rpo2 BspHI 1055-1117 fw	GGCCTCATGAGGACAATGATTTACGTTTGTGATC
FW683	*Saci_1342* upstream detection fw	GCGCATCTGTAGACGATGAA
FW684	*Saci_1342* downstream detection rv	GTGCAGCGAAATCAAAACAA
FW749	Promoter EF-1α C-less fw	CTTAAGTTCAAGGTAAACTTTTATTAACTCTATCACTATATCGGTTAAGAGAGATGGAAGGAGAATATAATTGAG
FW750	Promoter tRNA^{Leu} C-less fw	ATTTTTGTTGGAAAAACATATAAGGTAAAACTGAACTCAGTATAATAGATTGATGGAAGGAGAATATAATTGAG
FW733	*Sso* TFEβ C92S fw	GATGAAATTCCTTCCTTAAGTTGTAAAAGCTTAACC
FW734	*Sso* TFEβ C92S rv	CTTTTACAACTTAAGGAAGGAATTTCATCTAAACCA
FW735	*Sso* TFEβ C95S rv	GGTTAAGCTTTTAGAACTTAAGCAAGGAATTTCA
FW736	*Sso* TFEβ C95S fw	CCCTTGCTTAAGTTCTAAAAGCTTAACCAAATGTG
FW917	Promoter Rpo5 C-less fw	AGTAAGTCAGAGTAAAACTCTTATTAACCAAATGTAGTATGTGAATAATGGTTAATAATGAGAGGATATGGAAGGAGAATATAATTGAG
FW918	Promoter 16S/23S rRNA C-less fw	ATATAGAAGTTAGATTTATATGGGATTTCAGAACAATATGTATAATGGGGATTATGGAAGGAGAATATAATTGAG
FW925	Promoter SSB C-less fw	TTCATTAACACATAGATTTATAAATGGATAAAATGATATTTGAAATAAGAAATGTGAGATTGAAAGAGGGGAGGAAG
FW969	Promoter hybrid rRNA/ EF-1α C-less fw	ATATAGAAGTTAGATTTATATGGGATTTCAGAACACTATATCGGTTAAGAGAGATGGAAGGAGAATATAATTGAG
FW971	Promoter rRNA/tRNA^{Leu} C-less fw	ATATAGAAGTTAGATTTATATGGGATTTCAGAACACTCAGTATAATAGATTGATGGAAGGAGAATATAATTGAG
509	*Sso rpoG* NcoI fw	CCCCCCATGGTGGAATCAGTGGCACAAG
510	*Sso rpoG* BamHI rv	CCCCCGGATCCATTGTTTTTCTTCACGC

*restriction sites are underlined and ribosome-binding sites are depicted in bold.
