# Supplementary material for: Archaeal TFEα/β is a hybrid of TFIIE and the RNA polymerase III subcomplex hRPC62/39
Source: eLife. 2015 Jun 12;4:e08378. doi: 10.7554/eLife.08378 (PMC4495717; doi:10.7554/eLife.08378)
Supplement: Supplementary file 4. — List of in vitro transcription templates with promoters fused to a C-less cassette. DOI: http://dx.doi.org/10.7554/eLife.08378.025 [file elife08378s006.pdf]

Supplementary file 4 – List of *in vitro* transcription templates with promoters fused to C-less cassette

Plasmid ID	Backbone	Promoter	C-less cassette	Oligonucleotide pairs used for amplification
p1176	pGEM-T (Promega)	SSV1-T6	150 nt	FW642/FW643
p1205	pGEM-T (Promega)	Sso0216 (EF1	150 nt	FW749/FW643
p1206	pGEM-T (Promega)	SSOt30 (tRNA^{Leu})	149 nt	FW750/FW643
p1212	pGEM-T (Promega)	Rpo5	164 nt	FW917/643
p1213	pGEM-T (Promega)	16S/23S rRNA	150 nt	FW918/643
p1216	pGEM-T (Promega)	SSB	109 nt	FW925/643
p1236	pGEM-T (Promega)	hybrid rRNA/EF1α	150 nt	FW969/FW643
p1238	pGEM-T (Promega)	hybrid rRNA/tRNA^{Leu}	149 nt	FW971/FW643
